# Supplementary material for: Prediction of COVID-19 epidemic situation via fine-tuned IndRNN
Source: PeerJ Comput Sci. 2021 Nov 12;7:e770. doi: 10.7717/peerj-cs.770 (PMC8592248; doi:10.7717/peerj-cs.770)
Supplement: Supplemental Information 1 — Different strategies or events of China at different stages of the COVID-19 epidemic. [file peerj-cs-07-770-s001.docx]

| **Date** | **Stage** | **Event** |
| --- | --- | --- |
| Early December to mid-December 2019 | Occurrence stage | On December 1, 2019, the first confirmed case of COVID-19 developed symptoms (*Huang et al, 2020*). |
| Late December 2019 to mid-January 2020 | Development stage | 1. From December 26 to December 29, 2019, 7 patients with lung abnormalities appeared successively (*Human Resources and Social security Department of Hubei Province, 2020*); 2. Person-to-person transmission (*Li et al., 2020a*); 3. China CDC issued emergency response (L*i et al., 2020a*). 4. The number of cases increased, and the epidemic spread to other parts of China (N*ational Health Commission of the people's Republic of China, 2020*). |
| Late January to early March 2020 | Explosive stage | 1. At 10 o'clock on January 23, 2020, Wuhan was closed, and other areas were closed (*Hubei Provincial People's Government, 2020a*); 2. Initiation successive of first level response in various regions across the country (41. *People.cn., 2020*); 3. “Huoshenshan”, “Leishenshan”, Fangcang shelter hospitals in Wuhan were completed (*Chinanews, 2020*); 4. February 1, Prevention and control guidelines, Edition 1 (*Chinese Center for Disease Control and Prevention, 2020*). |
| Mid-March 2020 to April 28, 2020 | Recession stage | 1. The incident was under basic control; 2. Regions began to unseal, Wuhan officially unblocked on April 8, 2020 (*Hubei Provincial People's Government, 2020b*); |
| April 29, 2020 to April 24, 2021 | Continuous prevention and control | 1. As of May 2, 2020, all provinces across the country have lifted the first-level emergency response (*China Central Television, 2020*); 2. The promotion of resumption of work and school (*The State Council Information Office of the People’s Republic of China, 2020*). |
