# Supplementary material for: Prediction of COVID-19 epidemic situation via fine-tuned IndRNN
Source: PeerJ Comput Sci. 2021 Nov 12;7:e770. doi: 10.7717/peerj-cs.770 (PMC8592248; doi:10.7717/peerj-cs.770)
Supplement: Supplemental Information 2 — Different strategies or events of the United States at different stages of the COVID-19 epidemic. [file peerj-cs-07-770-s002.docx]

| **Date** | **Stage** | **Event** |
| --- | --- | --- |
| January 20, 2020 to February 23, 2020 | Occurrence stage | 1. On January 20, 2020, the first confirmed case was reported (*Holshue et al., 2020*); 2. During this time period, the US confirmed 14 local cases and 39 cases were withdrawn from overseas (*Jernigan, 2020*). 3. At 5:00 pm on February 2nd, Eastern Time, foreigners who have been to China in the past 14 days will be provisionally prohibited entry, while American citizens will be quarantined for 14 days (*National Immigration Administration, 2020*). |
| Late February 2020 to early March 2020 | Development stage | 1. Cases of travel history in epidemic-free areas appeared, the epidemic was gradually spreading in the US (*Jernigan, 2020*); 2. Mardi Gras celebrations, International professional conference in Boston, the funeral in Albany (*Schuchat, 2020*); 3. On March 14, Cruise ship ban (*Schuchat, 2020*). |
| Mid-March 2020 to April 24, 2021 | Explosive stage | 1. On April 3, the US CDC required residents to wear masks in public places (*Centers for Disease Control and Prevention, 2020*); 2. “Black Lives Matter” protest in parts of the US ( *Abcnews, 2020c*); 3. US President Trump held the first campaign rally in Tulsa (*Goodmorningamerica, 2020b*); 4. More U.S. states demand wearing masks in public (*Xinhuanet, 2020d*); 5. Trump called for wearing masks (*Xinhuanet, 2020e*); 6. Trump pushed reopening (*Goodmorningamerica, 2020a*); 7. In late August, many schools opened (*Xinhuanet, 2020f*); 8. Several presidential election rallies were held (*USA TODEY, 2020*). |
